# Inhibiting of TACC3 Promotes Cell Proliferation, Cell Invasion and the EMT Pathway in Breast Cancer

**DOI:** 10.3389/fgene.2021.640078

**Published:** 2021-06-03

**Authors:** Qin Huo, Siqi Chen, Zhenwei Li, Juan Wang, Jiaying Li, Ni Xie

**Affiliations:** ^1^Biobank, Institute of Translational medicine, Shenzhen Second People’s Hospital, Graduate School of Guangzhou Medical University, Shenzhen, China; ^2^Department of Clinical Medicine, University of South China, Hengyang, China

**Keywords:** TACC3, breast cancer, expression, cell proliferation, cell invasion

## Abstract

Accumulating evidences indicate that transforming acidic coiled-coil 3 (TACC3) is a tumor-related gene, was highly expressed in a variety of human cancers, which is involved in cancer development. However, the potential role of TACC3 in breast cancer remains largely unknown. In the present study, we found that TACC3 was highly-expressed in breast cancer tissues, and its level was positively correlated with the clinical features of breast cancer patients. Specifically, TACC3 expression was significantly associated with the estrogen receptor (ER), progesterone receptor (PR), human epidermal growth factor receptor 2 (HER2) status, nodal status, the scarff-bloom-richardson (SBR) grade, nottingham prognostic index (NPI), age, subtypes, and triple-negative and basal-like status, suggesting that TACC3 may be a potential diagnostic indicator of breast cancer. Furthermore, functional studies have shown that inhibition of TACC3 can significantly promote the cell proliferation and viability of breast cancer cells. Moreover, TACC3 knockdown suppressed the expression of E-cadherin, but increased the expression of N-cadherin, Snail, ZEB1, and TWIST, which indicate that TACC3 may impact the migration of breast cancer cells *in vitro*. Taken together, these findings indicate that TACC3 may serve as a prognostic and therapeutic indicator of breast cancer.

## Introduction

Breast cancer is the most common type of cancer in women around the world, and it is also one of the cancers with the highest mortality rates among women in the world (Bray et al., 2018). Metastasis is the primary cause of death in breast cancer patients (Huo et al., 2020). Although the current treatments have improved the survival rate and quality of life, the prognosis for patients with advanced cancer is still poor. Therefore, it is important to study the potential mechanism of cancer occurrence and development (Zeng et al., 2018).

The transforming acidic coiled-coil 3 (TACC3) belongs to the TACC family of centrosome proteins and has a highly conserved C-terminal curly coil domain (Lin et al., 2018). TACC3 is a tumor-related gene related to cancer development (Song et al., 2016). This gene product can regulate the formation of microtubules throughout the cell cycle (Kinoshita et al., 2005; Lioutas and Vernos, 2013). Studies have shown that TACC3 is highly expressed in lung cancer (Ha et al., 2015), bladder cancer (Wang et al., 2011), multiple myeloma (Stewart et al., 2004), osteosarcoma (Matsuda et al., 2018), prostate cancer (Li et al., 2017), glioblastoma (Singh et al., 2012), ovarian cancer (Lauffart et al., 2005), and colorectal cancer (Du et al., 2016). Additionally, previous studies demonstrated that TACC3 deficiency inhibited the epithelial-mesenchymal transition phenotype by activating the PI3K/Akt and ERK signaling pathways (Ha et al., 2013). Based on these studies, it seems that TACC3 may promote tumor progression by increasing cell proliferation, cancer stem cell population, and cancer cell migration (Ma et al., 2018). However, the potential role of TACC3 in breast cancer is still largely unknown.

In this report, we comprehensively analyzed the expression of TACC3 and correlated it with clinicopathological parameters of breast cancer patients by using extensive bioinformatics data mining process, and then analyzed the protein network of TACC3-predicted associations with and alterations in cancer genomics. Moreover, we investigated the mutation rate, single-nucleotide variation (SNV), copy number variation (CNV) distribution, and functional enrichment analyses of TACC3 in breast cancer. The results were validated by the experiments below. Knockdown of TACC3 increased cell migration of breast cancer *in vitro*. Taken together, these data indicate that TACC3 plays a role in the migration of and invasion by breast cancer cells *in vitro*.

## Results

### Retrieval of Significantly Dysregulated Genes in Breast Cancer

**FIGURE 1:**
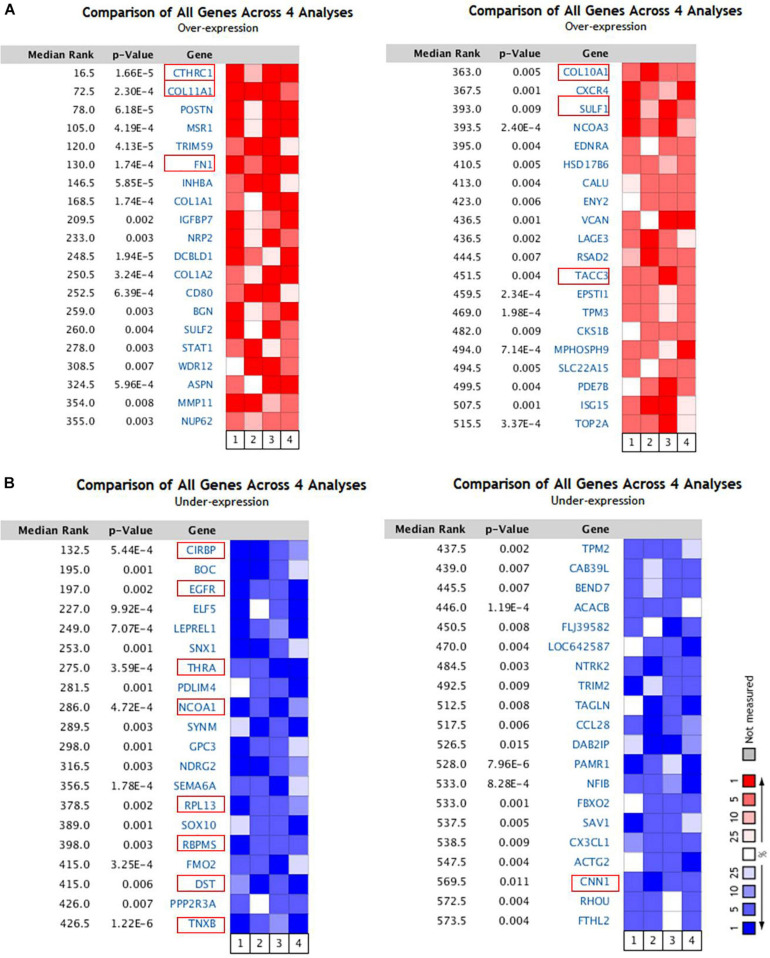
The 80 genes that were significantly dysregulated in breast cancer according to four independent microarrays retrieved from the Oncomine database. **(A)** The top 40 genes upregulated in microarrays. **(B)** The top 40 genes downregulated in microarrays.

To identify the genes related to the development and progression of breast cancer, we selected four independent microarrays stored in the Oncomine database. Based on the Oncomine online tool, all the genes from the data of four groups were compared. We discovered that 40 genes were significantly up-regulated (*p* < 9.00E-3) and 40 genes were significantly down-regulated (*p* < 1.50E-2) in breast cancer were retrieved (Figure 1). Over a total of 80 genes, 6 genes (CTHRC1, COL11A1, FN1, COL10A1, SULF1, and TACC3) were up-expressed (Figure 1A), and 9 genes (CIRBP, EGFR, THRA, NCOA1, RPL13, RBPMS, DST, TNXB, and CNN1) were down-expressed (Figure 1B).

### High Expression of TACC3 in Breast Cancer

**FIGURE 2:**
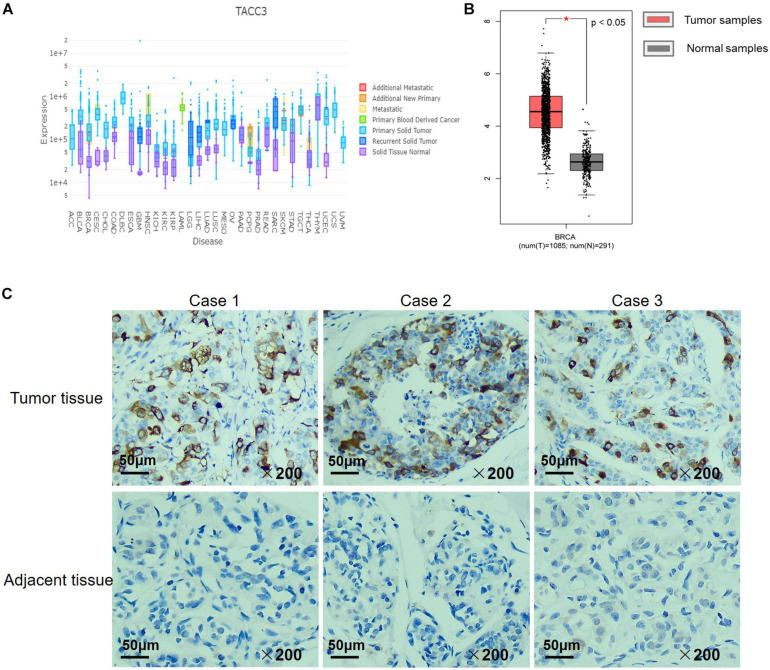
The TACC3 expression levels in different types of cancer and normal tissues. **(A)** The expression levels of TACC3 expression in different tumor types using DriverDB. Red represents up-regulated genes with log2 (fold change) > 1 and green represents down-regulated genes with log2 (fold change) < –1. Blue and purple dashed lines represent the average value of all the tumor and normal tissues, respectively. **(B)** Gene Expression Profiling Interaction Analysis (GEPIA) for the expression of TACC3 in breast tumor tissues and normal tissues. The TCGA data revealed that TACC3 mRNA was significantly higher in breast cancer tissues (1,085 cases) than that in normal tissues (291 cases). **(C)** Immunohistochemical staining revealed that TACC3 exhibited high expression in breast tumor tissues. The TACC3 proteins were detected with a TACC3-specific antibody (brown), Nuclei were stained with hematoxylin (blue). Fresh breast cancer tissue specimens were obtained from 30 patients who underwent surgery. Scale bar, 50 μm.

We examined the expression of TACC3 between breast tumors and adjacent normal tissues using the DriverDBv3 database. The results were shown in Figure 2A, and compared with normal tissues, the TACC3 transcription level was significantly higher in breast cancer. Additionally, to confirm the differences in TACC3 expression in breast cancer, the expression of TACC3 was analyzed by using RNA-seq data from multiple malignancies in TCGA. From the GEPIA database, we found that TACC3 mRNA in breast cancer tissues (1,085 cases) was significantly higher than that in normal tissues (112 cases) (*p* < 0.05) (Figure 2B). Furthermore, the representative immunohistochemical staining patterns for TACC3 were analyzed. We found that TACC3 was strongly expressed in breast cancer patient tissues (Figure 2C), but negative in adjacent tissues. Together, these results demonstrated that TACC3 was highly expressed in breast cancer patients.

### Relationship Between the TACC3 Expression and Clinical Characteristics of Breast Cancer Patients

**FIGURE 3:**
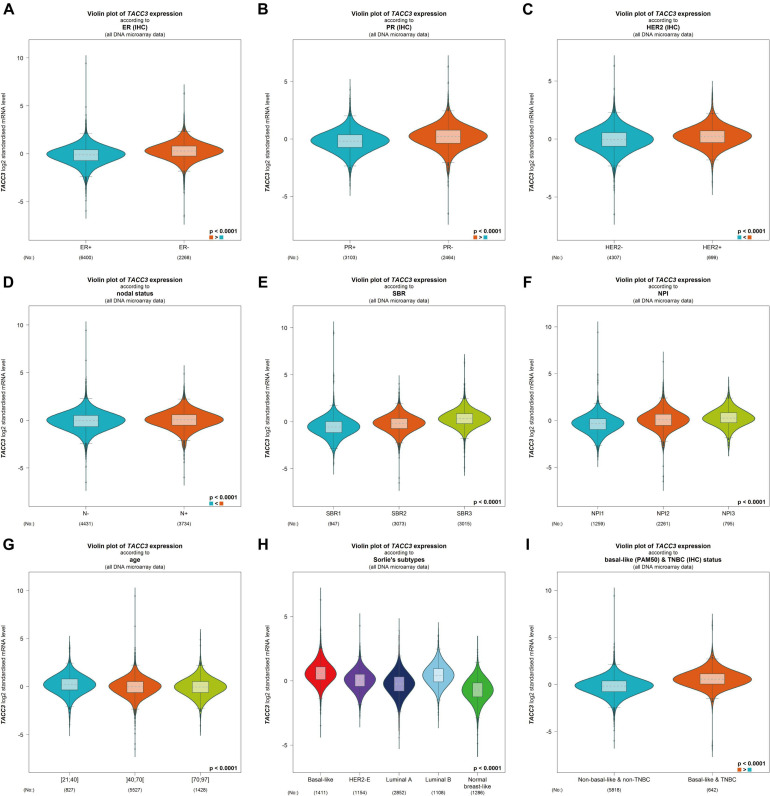
The relationship between the TACC3 expression levels in patients with breast cancer was addressed using bc-GenExMiner v4.4 based on different types of clinical characteristics parameters. **(A–C)** receptor status (ER + vs. ER -, PR + vs. PR -, HER2 + vs. HER2 -, **(D)** nodal status (N + vs. N-), **(E)** SBR (SBR1, SBR2, and SBR3), **(F)** NPI (NPI1, NPI2, and NPI3), **(G)** age (≤ 51 and > 51 years), **(H)** subtypes (basal-like, HER2-E, luminal A, luminal B, and normal breast-like), and **(I)** triple-negative status (TNBC vs. Not TNBC).

To better understand the correlation and potential mechanisms of TACC3 expression in breast cancer, we investigated the relationship between the TACC3 expression and clinical characteristics of breast cancer patients using bc-GenExMiner 4.4. The expression of TACC3 mRNA was significantly different in ER status (ER- > ER +, *p* < 0.0001, Figure 3A), PR status (PR- > PR +, *p* < 0.0001, Figure 3B), and HER2 status (HER2 + > HER2-, *p* = 0.0006, Figure 3C). In addition, TACC3 expression was significantly increased in patients with a negative nodal status (N+) than those with positive nodal status (N−) (*p* < 0.001) (Figure 3D). The analysis of SBR grade showed that the increased SBR level was significantly associated with the increased TACC3 transcript level (SBR1 < SBR2 < SBR3, *p* < 0.001) (Figure 3E). The NPI grade showed a consistent trend (NPI1 < NPI2 < NPI3, *p* < 0.001) (Figure 3F). For patient age, TACC3 mRNA expression was significantly higher in the 21–40 year group than in the 41–70 years group and 70–97 years groups (*p* < 0.001, Figure 3G). In addition, we found that there were significant difference in the expression of TACC3 mRNA in the subtypes (basal-like > luminal B > HER2E > luminal A > normal breast-like, *p* < 0.001, Figure 3H) and basal-like and TNBC status (basal-like and TNBC > Non-basal like and non-TNBC, Figure 3I). These results suggest that TACC3 expression may serve as a potential diagnostic indicator in breast cancer.

### Analysis of the Mutation Rate, CNVs Distribution, and Functional Enrichment Analyses of TACC3 in Breast Cancer

**FIGURE 4:**
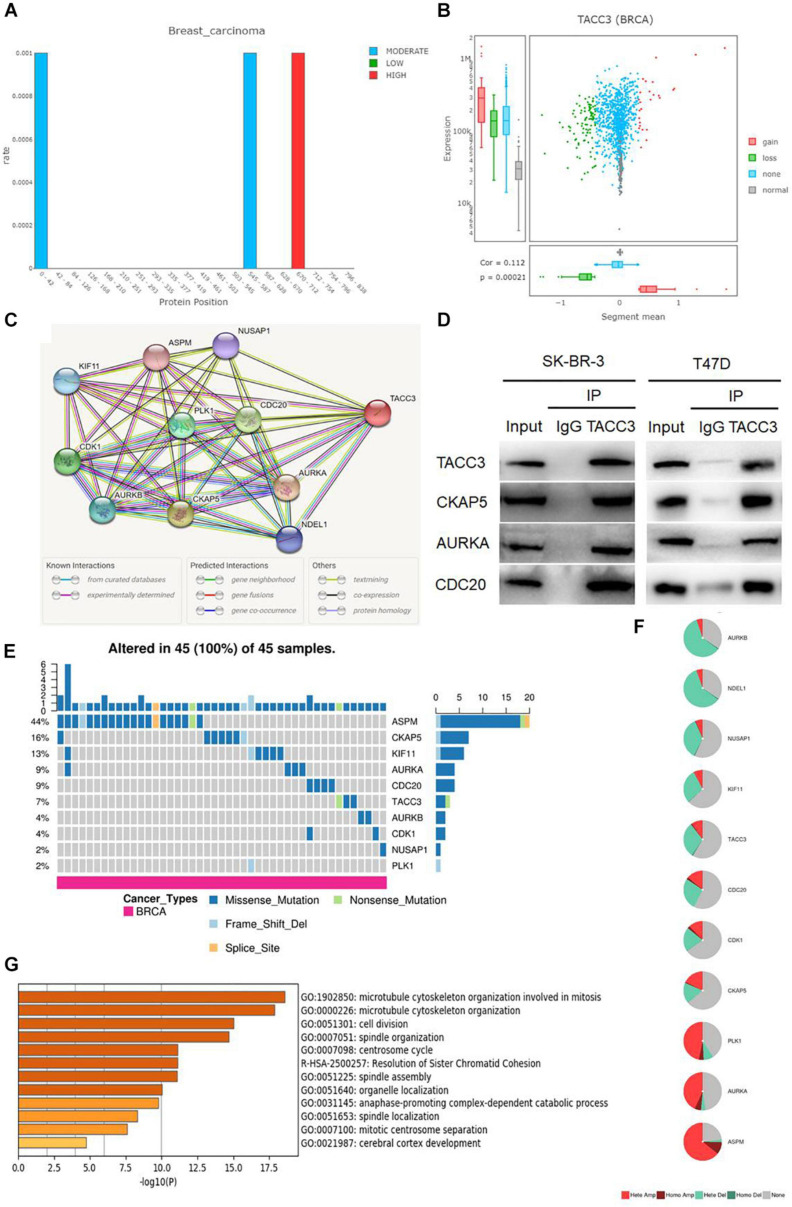
Analysis of the mutation rate, CNVs distribution, and functional enrichment analyses of TACC3 in breast cancer. **(A)** the mutation rate of TACC3 and its protein positions in breast cancer. The mutation squares indicate the number of mutation tools that identify this gene as a mutation driver. **(B)** The CNV squares indicated CNV gain or loss of TACC3 in breast cancer. The red represents a gain (1) and the green represents a loss (–1). **(C)** The PPI network of TACC3 was constructed using the STRING database, and the network of TACC3 and its co-expression genes were set up visually. **(D)** Immunoprecipitation of anti TACC3 antibodies with CKAP5, AURKA, and CDC20 in cells. **(E,F)** Single-nucleotide variation (SNV) and copy number variation (CNV) analysis by GSCALite. **(G)** Metascape enrichment analysis results on the interacting genes that interacted with TACC3.

We used the DriverDBv3 tool to determine the mutation rate and CNVs distribution of TACC3 in breast cancer. Figure 4A shows the mutation rate of TACC3 and its protein positions in breast cancer. We found that the TACC3 protein had the highest mutation rate at positions 670–671. On the contrary, the mutation rate of the TACC3 protein was the lowest at 0–42 and 545–587. Additionally, the CNVs squares indicate the CNVs gain or loss for TACC3 in breast cancer (Figure 4B). We found that the distribution of CNVs primarily included gain, loss, none, and normal, and was positively correlated with the expression of TACC3 in breast cancer (cor = 0.112, *p* = 0.0021).

To validate the biological interaction network of TACC3 in breast cancer, we identified genes co-expressed with TACC3 using the STRING database (Figure 4C). The following ten proteins were found to interact with TACC3: cytoskeleton-associated protein 5 (CKAP5), aurora kinase A (AURKA), cell division cycle protein 20 homolog (CDC20), cyclin-dependent kinase 1 (CDK1), serine/threonine-protein kinase PLK1 (PLK1), aurora kinase B (AURKB), kinesin-like protein KIF11 (KIF11), nuclear distribution protein nudE-like 1 (NDEL1), nucleolar and spindle associated protein 1 (NUSAP1), and abnormal spindle-like microcephaly-associated protein (ASPM), and their correlation scores were 0.999, 0.999, 0.974, 0.971, 0.970, 0.968, 0.964, 0.962, 0.956, and 0.955, respectively. As shown in the Figure 4D, the top three proteins (CKAP5, AURKA, and CDC20) interacting with TACC3 were verified by immunoprecipitation in breast cancer cells, and CKAP5, AURKA, and CDC20 were detected in the immunoprecipitation of anti TACC3 antibody. This result indicates that TACC3 physically interacts with these proteins, and the potential biological function of TACC3.

To further understand the SNVs and CNVs of these proteins, we performed an analysis with GSCALite (Figures 4E,F). We found that the SNVs frequencies of ASPM, CKAP5, KIF11, AURKA, CDC20, and TACC3 were in the top six, at 44, 16, 13, 9, 9, and 7%, respectively. Among them, the variant types of TACC3 were primarily missense mutations. The metascape enrichment analysis results on the TACC3 interacting genes are shown in Figure 4G. The results showed that the biological processes of these genes were enriched in the microtubule cytoskeleton organization involved in mitosis, microtubule cytoskeleton organization, and cell division. Therefore, we hypothesized that TACC3 might serve as a biomarker for breast cancer research.

### TACC3 Knockdown Promotes Tumor Formation

**FIGURE 5:**
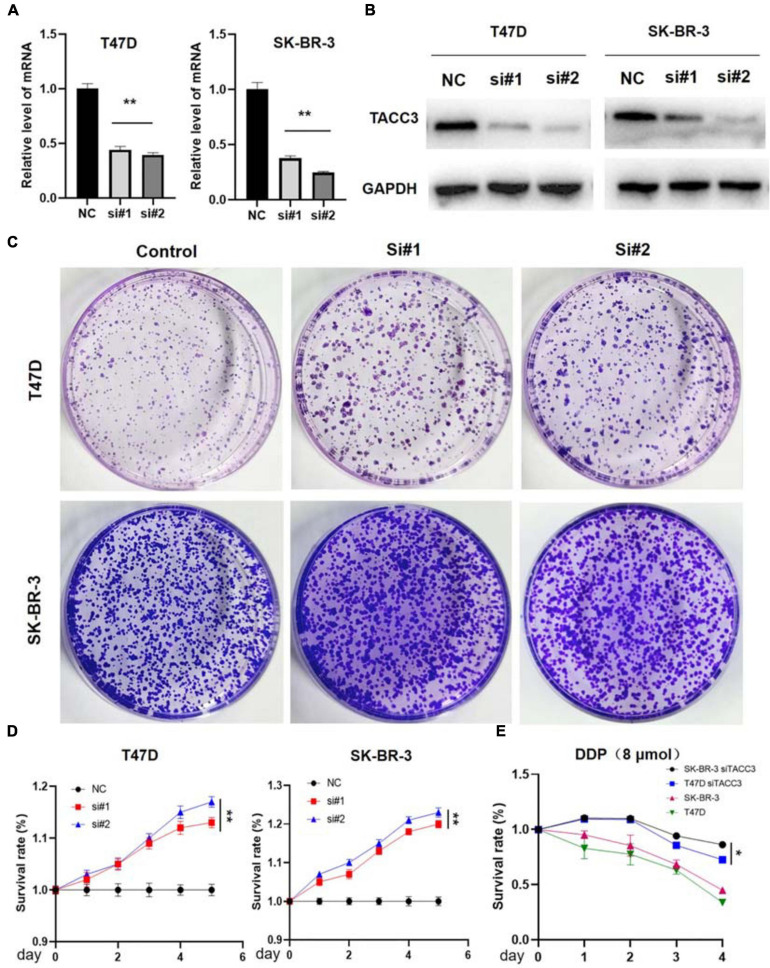
TACC3 knockdown promotes tumor formation in the T47D and SK-BR-3 cell lines. **(A)** Verification of TACC3 in T47D and SK-BR-3 cells by quantitative RT-PCR. **(B)** Verification of TACC3 in T47D and SK-BR-3 cell lines by quantitative Western blot analysis. **(C)** Colony formation assay for assessing the cell proliferation of siTACC3-treated cells. **(D)** Growth curves of T47D and SK-BR-3 cell before and after TACC3 gene knockdown. **(E)** The effect of DDP 8 μmol on the viability of breast cancer cells. All assays were performed in biological triplicate. Data are shown as mean ± S.E.M. **compared with NC, *p* < 0.01. *compared with NC, *p* < 0.05. *P*-values are calculated by Student’s *t*-test.

TACC3 is highly expressed in breast cancer, but whether the downregulation of TACC3 represses tumorigenesis remains unknown. Therefore, the effect on colony proliferation and cell formation can be observed in the TACC3-knockdown T47D and SK-BR-3 cells by using siRNA (Figures 5A,B). Furthermore, we explored the effects of TACC3 expression on cell proliferation and cell colony-forming ability, the effect of TACC3 on cell proliferation can be demonstrated by the CCK8 and colony formation assays (Figure 5C). The CCK8 cell proliferation assays results showed that TACC3 knockdown in T47D and SK-BR-3 cells increased cell capacity compared with the NC groups (Figure 5D). Notably, similar results can be observed from the colony formation assays, the cell colony-forming number increased in the TACC3-knockdown T47D and SK-BR-3 cells. Meanwhile, we treated cells with cytotoxic drug cisplatin (DDP) and found that the survival rate of cells with knockdown TACC3 was significantly higher compared with negative control cells by CCK8 assay (Figure 5E). We hypothesized that the cells with knockdown of TACC3 had enhanced resistance to apoptosis from cell injury.

### TACC3 Inhibited Breast Cancer Cell Migration *in vitro*

**FIGURE 6:**
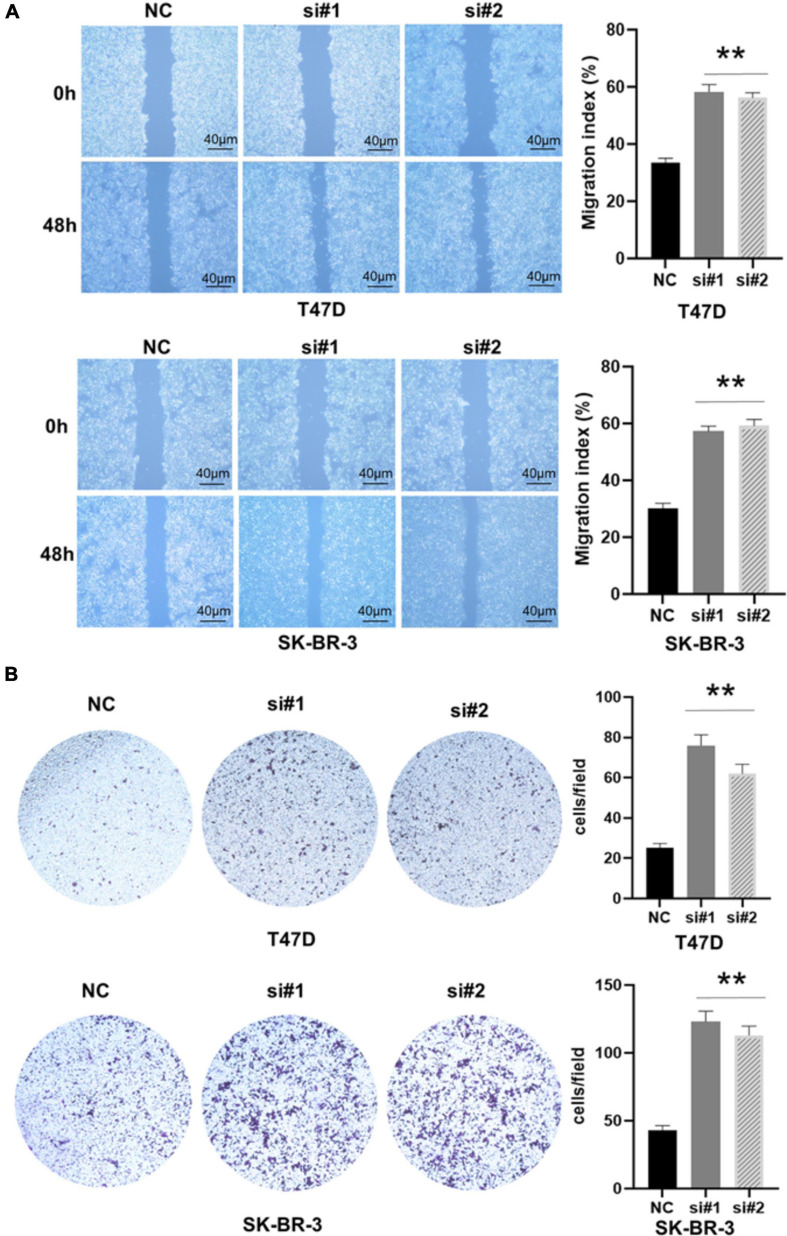
TACC3 knockdown promotes the migration and invasion of the T47D and SK-BR-3 cell line. **(A,B)** Classic wound healing assay for assessing the migration of siTACC3-treated cells. **compared with control, *p* < 0.01. All assays were performed in biological triplicate. Data are shown as mean ± S.E.M. **compared with NC, *p* < 0.01. *P*-values are calculated by Student’s *t*-test.

Previous studies have shown that EMT is related to the occurrence, invasion, and metastasis of tumors (Pastushenko and Blanpain, 2019). Because TACC3 knockdown can promote cell invasion of breast cancer, we speculate that TACC3 can affect the cell EMT process in breast cancer. To determine the role of TACC3 in breast cancer metastasis, we first transfected the T47D and SK-BR-3 cell lines with the control vector and TACC3 under-expression vector. Cell migration to the wound area was analyzed at 0 h and 48 h after injury. The results showed that knocking down TACC3 significantly promoted wound healing compared with the negative control (Figure 6A). The transwell invasion assays results showed that knockdown of TACC3 in T47D and SK-BR-3 cell lines increased cell invasion number compared with the si-NC groups (Figure 6B). Taken together, these data indicate that TACC3 plays a role in the migration and invasion of breast cancer cells *in vitro*.

### TACC3 Might Be Involved in Notch4 and CDH5-Related Signaling Pathways

**FIGURE 7:**
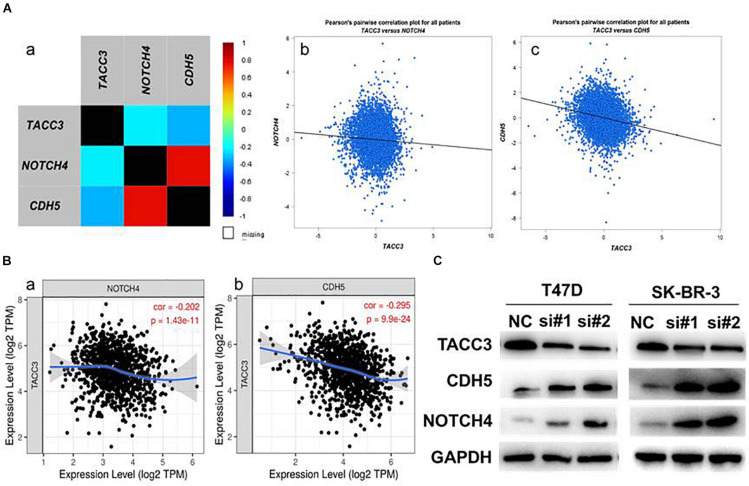
The expression correlation between TACC3 and Notch4 and CDH5. **(A)** The correlation between TACC3 and Notch4 and CDH5 according to bc-GenExMiner v 4.4. **(B)** The correlation between TACC3 and Notch4 and CDH5 according to the TIMER database. **(C)** The Notch4 and CDH5 protein levels were evaluated by Western blot analysis in the siTACC3-treated cells. All assays were performed in biological triplicate.

Notch4 and CDH5 are two important genes involved in EMT and the aggressive phenotype of breast cancer (D’Angelo et al., 2015; Fry et al., 2016). To verify the correlation between TACC3 and Notch4 and CDH5, we examined the RNA-seq data (*N* = 4,712) of breast cancer cohort in the TCGA database by using bc-GenExMinerv 4.4 (Figure 7A). The results showed that TACC3 expression was negatively correlated with Notch4 (*r* = −0.23, *p* < 0.0001) and CDH5 (*r* = −0.33, *p* < 0.0001). Then, we further investigated the results using the TIMER database (Figure 7B). We found that TACC3 expression was negatively correlated with Notch4 (*r* = −0.202, *p* = 1.43e-11) and CDH5 (*r* = −0.295, *p* = 9.9e-24). Moreover, we used western blotting to study the changes of protein markers in TACC3-knockdown breast cancer cells. The result was consistent with our hypothesis that knockdown of TACC3 in T47D and SK-BR-3 cells resulted in increased expression of Notch4 and CDH5 (Figure 7C). These findings suggest that TACC3 may be closely related to the Notch4 and CDH5 signaling pathways in breast cancer.

### TACC3 Represses the Epithelial-Mesenchymal Transition Through the Wnt/β-Catenin and Notch Signaling Pathways

**FIGURE 8:**
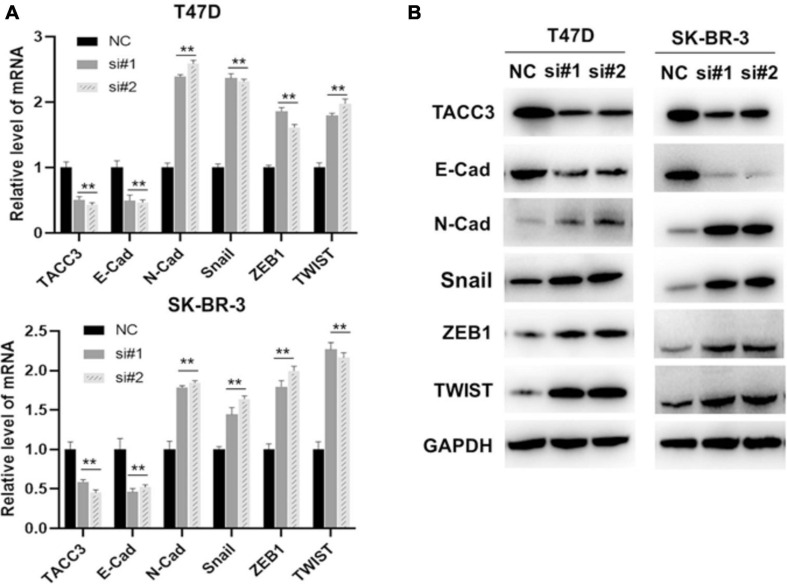
TACC3 represses the epithelial-mesenchymal transition (EMT) through the Wnt/β-catenin and Notch signaling pathways. **(A)** RT-PCR assay for assessing the expression levels of E-cadherin, N-cadherin, Snail, ZEB1, and TWIST following siTACC3-treatment. **(B)** Western blot analysis assay on the expression of EMT-related factors in siTACC3-treated cells. All assays were performed in biological triplicate. Data are shown as mean ± S.E.M. ***Compared with NC cells, *p* < 0.001; **compared with NC cells, *p* < 0.01; *compared with NC cells, *p* < 0.05. *P*-values are calculated by Student’s *t*-test.

Our previous studies have shown that the Notch4 and CDH5 activity are activated by siTACC3, and hypothesized that the loss of TACC3 may also promote EMT through the Wnt/β-catenin and Notch signaling pathways. To examine this hypothesis, the expression of E-cadherin, N-cadherin, Snail, ZEB1, and TWIST were analyzed after siTACC3 treatment. The RT-PCR analysis revealed that knocking of TACC3 in the T47D and SK-BR-3 cells decreased the expression of epithelial marker E-cadherin, but increased the expression of mesenchymal markers N-cadherin, Snail, ZEB1, and TWIST (Figure 8A). We then used western blots to study the changes of EMT protein markers after TACC3 knockdown in breast cancer cells (Figure 8B). This result is consistent with our hypothesis that the stromal markers that promote metastasis (such as N-cadherin, Snail, ZEB1, and TWIST) are up-regulated, while the epithelial markers that inhibit metastasis (E-cadherin) are down-regulated. These results suggest that TACC3 plays an important role in breast cancer metastasis.

## Discussion

TACC3 is a member of the TACC family located on human chromosome 4p16 (Song et al., 2016). TACC3 is one of the important regulators of microtubule stability in the process of mammalian mitotic spindle assembly (Lin et al., 2018). TACC3 also plays an important role in transcription and tumorigenesis (Guo and Liu, 2018). Zhi-Rui Lin et al. found that the up-regulation of TACC3 was positively correlated with the tumor aggressiveness, grade, T stage, and progression in bladder cancer patients (Lin et al., 2018). The expression of TACC3 may predict the radiosensitivity and prognosis of locally advanced rectal cancer (Ma et al., 2018). In addition, TACC3 can be used as a biomarker with a potential role in breast cancer detection and for the prediction of clinical outcomes (Campo and Breuer, 2018). Despite these observations, the potential role of TACC3 and its molecular mechanisms in breast cancer remains to be addressed. In the present study, we found that the expression level of TACC3 was significantly higher in breast cancer tissues than that in normal tissues, which is consistent with Song et al. (2016).

Furthermore, to better understand the relevance and potential mechanisms of TACC3 expression in breast cancer, we investigated the relationship between the TACC3 expression and clinical characteristics of breast cancer patients and found that TACC3 expression was significantly associated with the ER, PR, HER2 status, nodal status, SBR, NPI, age, subtypes, and triple-negative and basal-like status. These results suggest that TACC3 expression may be a potential diagnostic indicator for breast cancer. In addition, the protein-protein interaction data demonstrated that CKAP5, AURKA, and CDC20 showed the highest correlation with TACC3. AURKA has been confirmed as an oncogene in cancer development (Nikonova et al., 2013). Furukawa T et al. revealed that AURKA was one of the downstream targets of MAPK1/ERK2 in pancreatic cancer (Furukawa et al., 2006). CDC20 is associated with malignant progression and poor prognosis among various cancers (Cheng et al., 2019). It may be a promising therapeutic target against human cancer (Wang et al., 2015). Therefore, the close interaction of TACC3 with CKAP5, AURKA, and CDC20 may suggest the role of TACC3 in the progression of breast cancer, although future mechanistic studies are needed to verify this possibility. We performed SNV and CNV analyses of these proteins using GSCALite, and the variant type of TACC3 was primarily missense mutation. In addition, the enrichment analysis of TACC3 interaction showed that the biological processes of these genes were enriched in microtubule cytoskeleton organization involved in mitosis, microtubule cytoskeleton organization, and cell division. These results reveal the potential regulatory role of TACC3 in breast cancer.

A great deal of effort has been expended on studying the biological mechanisms of TACC3 over-expression in cancers (Stewart et al., 2004; Lauffart et al., 2005; Wang et al., 2011; Singh et al., 2012; Ha et al., 2015; Du et al., 2016; Li et al., 2017; Matsuda et al., 2018). However, little is known about the precise mechanism of TACC3 downregulated. Therefore, The effects on colony formation and cell proliferation can be observed on TACC3-knocked down in T47D and SK-BR-3 cells. To determine the role of TACC3 in breast cancer metastasis, we transfected T47D and SK-BR-3 cell lines with the control vector and TACC3 under-expression vector. Inhibition of TACC3 expression by siRNA could significantly promote cell proliferation and viability. Taken together, these data indicate that TACC3 plays a role in the migration and invasion of breast cancer cells *in vitro*. Notch4 and CDH5 are two important genes involved in EMT and the aggressive phenotype of breast cancer (D’Angelo et al., 2015; Fry et al., 2016). We also investigated the possible relationship between TACC3, Notch4, and CDH5 in the proliferation of breast cancer cells. Many studies have proved that the high expression of Notch4 and CDH5 is an important factor to promote tumor development (Soriano et al., 2000; Berx and van Roy, 2009; Mao et al., 2013; Giuli et al., 2019). We have verified the significant negative correlation between TACC3 and these two proteins through public databases and experiments. Knockdown of TACC3 upregulates the expression of Notch4 and CDH5, suggesting that TACC3 may be closely related to Notch4 and CDH5 signaling pathways in breast cancer.

According to reports, TACC3 reported being a negative regulator of Notch signaling by binding to the CDC10/anchorin repeat sequence (Bargo et al., 2010). Previous studies have found that TACC3 deficiency can inhibit the epithelial-mesenchymal transition phenotype by activating the PI3K/Akt and ERK signaling pathways, resulting in a high apoptosis rate of p53-mediated (Piekorz et al., 2002; Ha et al., 2013). Therefore, we hypothesized that the loss of TACC3 may also promote EMT through the Wnt/β-catenin and Notch signaling pathways. To examine this hypothesis, the expression of E-cadherin, N-cadherin, Snail, ZEB1, and TWIST were analyzed after siTACC3 treatment. The downregulation of TACC3 inhibited the expression of E-cadherin and increased the expression of N-cadherin, Snail, ZEB1, and TWIST. These results suggest that TACC3 plays an important role in the metastasis of breast cancer.

## Materials and Methods

### Data Source

The Oncomine database^1^ is the largest oncogene chip database platform in the world. It has a collection of 715 gene expression datasets, for s total of 86,733 samples of cancer tissues and normal tissues samples. It can be used to mine oncogene-related information (Rhodes et al., 2004). Initially, 12 datasets were found when we used the following filters: (a) analysis type: differential analysis, cancer versus normal analysis; (b) cancer type: breast cancer, invasive breast carcinoma-luminal-like subtype of invasive breast carcinoma; and (c) dataset filters: data type, mRNA. To retrieve stable and consistently mismatched genes in breast cancer, we then selected four studies from 12 datasets according to the following criteri (A) (a) breast carcinoma versus normal; (b) data type: mRNA; and (c) microarray platform is Human Genome U133 or U133 Plus 2.0. According to four microarray studies (1. invasive ductal breast carcinoma stroma vs. normal Karnoub breast, Nature, 2007; 2. ductal breast carcinoma vs. normal Richardson breast 2, Cancer Cell, 2006; 3. invasive ductal breast carcinoma vs. normal Turashvili breast, BMC Cancer, 2007; and 4. invasive lobular breast carcinoma vs. normal Turashvili breast, BMC Cancer, 2007), the genes with obvious disorders in breast cancer tissues were identified.

### TACC3 Expression Analysis

The expression of TACC3 in different cancer types was investigated using the DriverDB database^2^, which is a database of cancer histology, including the annotation basis, somatic mutation, RNA expression, miRNA expression, methylation, copy number variation, and clinical data (Liu et al., 2020). We then used the GEPIA database^3^ to analyze the expression level of TACC3 in breast cancer tissues and normal tissues (Tang et al., 2017).

### Association of Clinicopathological Parameters With TACC3 in Breast Cancer Patients

bc-GenExMinerv 4.4 is a database containing the published annotated genomic data, including data from DNA microarrays (*n* = 10,012) and RNA-seq (*n* = 4,713) (Jézéquel et al., 2012). We analyzed the TACC3 expression levels based on various classification parameters, such as the estrogen receptor (ER) and progesterone receptor (PR) expression, HER2 status (ER + / ER-, PR + / PR-, and HER2 + /HER2-), nodal status (N + /N-), scarff-bloom-richardson grade (SBR: SBR1, SBR2, and SBR3), nottingham prognostic index (NPI: NPI1, NPI2, and NPI3), age (21–40, 40–70, and 70–97), subtypes (basal-like, luminal B, HER2E, luminal A, and normal breast-like), and triple-negative and basal-like status (triple-negative and TNBC and not basal-like status and not TNBC). A value of *p* < 0.05 was considered statistically significant.

### Bioinformatic Analyses

We used the DriverDBv3 tool to determine the mutation rate and CNVs distribution of TACC3 in breast cancer (Berx and van Roy, 2009). Next, we analyzed the functional protein association network using the STRING database^4^ (Crosara et al., 2018). In addition, we performed the SNVs and CNVs of these proteins analyses with Gene set cancer analysis (GSCALite:^5^) (Akbani et al., 2014). We analyzed the gene set for SNVs using the statistics, distribution, and types; for the CNVs, we analyzed the statistics of deletion/amplification for hetero/homozygous CNVs. The pathway and process enrichment analysis were performed with metascape (Zhou et al., 2019).

### Cell Culture, Reverse Transfection, and Quantitative PCR Analyses

Breast cancer cell lines including T47D and SK-BR-3 cells were obtained from the Cell Bank of the Chinese Academy of Sciences (Shanghai, China). All of the cells were cultured in RPMI Medium 1640 supplemented with 10% fetal bovine serum (FBS) (GIBCO, United States). SK-BR-3 cells were cultured in DMEM supplemented with 10% FBS.

siRNAs were purchased from GenePharma (Suzhou, China). Sequences of si#1 were 5′-GCAGGUCUUAAACGACAAATT, sequences of si#2 were 5′-ACTAGATTGCTTTGAAAAC. The cells were transfected with TACC3 siRNA using Lipofectamine 2000 (Invitrogen, United States). The cells were harvested 72 h after transfection. The total cellular RNA was extracted using a PureLink RNA Mini Kit (Invitrogen, United States), and the RNA quality was examined by Nanodrop (Thermo, United States) analysis. The total mRNA was reverse-transcribed into cDNA using the 5 × Primescript RT Master Mix (Takara). Quantitative RT-PCR was performed using 2 × TB Green Premix Ex Taq (Takara) in a Bio-Rad detection system. The data for each gene was normalized against the β-actin levels.

### Western Blotting and Immunoprecipitation

The T47D and SK-BR-3 cells were homogenized in RIPA lysis buffer (Meilunbio, Shanghai, China) containing protease inhibitor mixture on ice for 30 min, then centrifuged for 10 min at 12,000 rpm in a 4°C Eppendorf microfuge (Thermo, United States). Loading buffer was added to the protein lysate and boiled for 5 min. The samples were then separated on SDS polyacrylamide gels, which were transferred to PVDF membranes (Millipore) and probed with their respective antibodies. The following primary antibodies were used: TACC3 (1:1,000; Abcam, United States), CDH5 (1:1,000, Abcam, United States), Notch4 (1:1,000, Abcam, United States), E-cadherin (1:1,000, Cell Signaling Technology, United States) and N-cadherin (1:1,000, Cell Signaling Technology, United States), Snail (1:1,000, Cell Signaling Technology, United States), ZEB1 (1:1,000, Abcam, United States), TWIST (1:1,000, Abcam, United States), and GAPDH (1:1,000, Abcam, United States).

For immunoprecipitation, cells were homogenized in IP lysis buffer (Meilunbio, Shanghai, China) containing protease inhibitor mixture on ice for 30 min. An antibody specific for the target protein was incubated with Protein G-agarose beads (Invitrogen, United States) for 4 h at 4°C, and the lysate was then added to the mixture. The mixture was incubated at 4°C for 16 h. After being washed three times with ice-cold PBS, beads-bound IP product was collected, and proteins were analyzed by western blotting.

### Colony Formation and Cell Proliferation Assay

A total of 200 cells were seeded into a 35 mm cell culture dish and cultured for 15 days in RPMI 1,640 medium (Gibco) supplemented with 10% FBS (Gibco). The colonies were imaged and counted after being fixed with 4% paraformaldehyde and stained with 1% crystal violet solution. NC and T47D siTACC3 cells were seeded into each well of 96 well plates, with the density of 4 × 10^3^ cells in 100 μL 1,640 medium (Gibco), and were grown overnight. The medium was then replaced with 100 μL 1,640 medium with 10% CCK8 reagent and incubated for 3 h at 37°C. At 0, 1, 2, 3, 4, and 5 days, the absorbance was measured at 450 nm.

### Wound Healing and Transwell Invasion Assays

For the wound healing assay, NC and siTACC3 cells were cultured in six-well plates coated with 0.1% gelatin. When 70% confluency is reached, the cells were starved overnight, a wound was scratched into the center of the cell monolayer with a sterile plastic pipette tip, and the debris was removed by washing with PBS washing. The wound was photographed at the indicated time.

For the transwell invasion assays, NC cells or siTACC3 cells were prepared as a cell suspension using a serum-free medium, we seeded 1 × 10^5^ cells in the upper chamber of the transwell plate. A complete medium containing 10% FBS was added to the lower chambers. Culture in a cell culture incubator for 24 h. The migrated cells in the lower chamber were fixed with 4% paraformaldehyde, stained with crystal violet for 10 min, and then analyzed by optical microscope.

### Immunohistochemistry Analysis

This study was performed on archived tissues from 30 diagnosed cases of breast cancer. Breast cancer tissue samples were taken from breast cancer patients who underwent surgery in Shenzhen Second People’s Hospital from January 2018 to December 2019. All the patients signed the informed consent form. This study was approved by the Ethics Committee of Shenzhen Second People’s Hospital in accordance with the principles of the Declaration of Helsinki. First, the collected tissue samples were embedded, sliced, and dewaxed; then performed antigen repair was performed as follows: prepared 200 mL of 10 × repair solution, such as 20 mL 10 × repair solution + 180 mL distilled water was prepared, each slice was placed in the repair box and close the lid was closed, the box was placed in a 100°C water bath and reacted for 20 min, then naturally cooled to room temperature; then, it was incubated in 3% H_2_O_2_ deionized water at room temperature in the dark for 10 min and rinsed with distilled water 2–3 times, for 3 min each time. The water around the slices was wiped off with filter paper, draw a circle of oil was drawn, and add an appropriate amount of blocking solution was added for protein blocking and the slices were incubated at room temperature for 20 min. The tissue was then incubated with the primary antibody and incubated overnight at 4°C, and then incubated with the secondary antibody. Avidin was added, incubated at room temperature in the dark for 20 min, then washed with PBST, followed by DAB color development + hematoxylin counterstaining and dehydration. Lastly, an image was taken with a scanning microscope.

### Statistical Analysis

Statistical analysis was performed using SPSS 25.0 (SPSS, Inc., Chicago, IL, United States). Data were presented as the means of the results from at least three independent experiments. one-way analysis of variance F(ANOVA) was used to process, and Student’s *t*-test was employed to assess significant differences. A threshold of *p* < 0.05 was defined as significant for all tests.

## Conclusion

In conclusion, we found that TACC3 expression was upregulated in breast cancer tissues. TACC3 expression was positively associated with the clinical characteristics of breast cancer patients. Furthermore, we demonstrated that the TACC3 knockdown promoted cell proliferation, cell invasion ability, and EMT signaling pathways in breast cancer. The mechanism of TACC3 in breast cancer requires further study. Thus, these results indicate that TACC3 has important clinical and therapeutic value in breast cancer. Our results suggest that TACC3 may serve as a potential target of breast cancer treatment.

## Data Availability Statement

Publicly available datasets were analyzed in this study. This data can be found here: Oncomine database (https://www.oncomine.org/resource/login.html), DriverDB database (http://driverdb.tms.cmu.edu.tw/), GEPIA database (http://gepia.cancer-pku.cn/index.html), bc-GenExMiner v4.4 (http://bcgenex.centregauducheau.fr/), STRING database (https://string-db.org/), and GSCALite datebase (http://bioinfo.life.hust.edu.cn/web/GSCALite/).

## Ethics Statement

The studies involving human participants were reviewed and approved by The Ethics Committee of Shenzhen Second People’s Hospital. The patients/participants provided their written informed consent to participate in this study.

## Author Contributions

QH designed the work and wrote the manuscript. SC and ZL participated in data analysis and participated in the methods. JW and JL performed part of the experiments. NX reviewed the manuscript. All authors contributed to the article and approved the submitted version.

## Conflict of Interest

The authors declare that the research was conducted in the absence of any commercial or financial relationships that could be construed as a potential conflict of interest.

## Footnotes

1 https://www.oncomine.org/resource/login.html

2 http://driverdb.tms.cmu.edu.tw/

3 http://gepia.cancer-pku.cn/index.html

4 https://string-db.org/

5 http://bioinfo.life.hust.edu.cn/web/GSCALite/
